# “The balancing act”— Licensed practical nurse experiences of falls and fall prevention: a qualitative study

**DOI:** 10.1186/1471-2318-12-62

**Published:** 2012-10-15

**Authors:** Beatrice Häggqvist, Michael Stenvall, Anncristine Fjellman-Wiklund, Kristina Westerberg, Lillemor Lundin-Olsson

**Affiliations:** 1 Department of Community Medicine and Rehabilitation, Physiotherapy, Umeå University, SE-90187, Umeå, Sweden; 2 Department of Community Medicine and Rehabilitation, Geriatric Medicine, Umeå University, SE-90187, Umeå, Sweden; 3 Department of Psychology, Umeå University, SE-90187, Umeå, Sweden; 4 Physiotherapy, Caring Sciences Building, Umeå University, SE-90187, Umeå, Sweden

**Keywords:** Accidental falls/*prevention&control, Licensed practical nurses, Focus groups, Safety culture

## Abstract

**Background:**

Falls are common in old age and may have serious consequences. There are many strategies to predict and prevent falls from occurring in long-term care and hospitals. The aim of this study was to describe licensed practical nurse experiences of predicting and preventing further falls when working with patients who had experienced a fall-related fracture. Licensed practical nurses are the main caretakers that work most closely with the patients.

**Methods:**

A qualitative study of focus groups interviews and field observations was done. 15 licensed practical nurses from a rehabilitation ward and an acute ward in a hospital in northern Sweden were interviewed. Content was analyzed using qualitative content analysis.

**Results:**

The result of the licensed practical nurse thoughts and experiences about risk of falling and fall prevention work is represented in one theme, “the balancing act”. The theme includes three categories: “the right to decide”, “the constant watch”, and “the ongoing negotiation” as well as nine subcategories. The analysis showed similarities and differences between rehabilitation and acute wards. At both wards it was a core strategy in the licensed practical nurse work to always be ready and to pay attention to patients’ appearance and behavior. At the rehabilitation ward, it was an explicit working task to judge the patients’ risk of falling and to be active to prevent falls. At the acute ward, the words “risk of falling” were not used and fall prevention were not discussed; instead the licensed practical nurses used for example “dizzy and pale”. The results also indicated differences in components that facilitate workplace learning and knowledge transfer.

**Conclusions:**

Differences between the wards are most probably rooted in organizational differences. When it is expected by the leadership, licensed practical nurses can express patient risk of falling, share their observations with others, and take actions to prevent falls. The climate and the structure of the ward are essential if licensed practical nurses are to be encouraged to routinely consider risk of falling and implement risk reduction strategies.

## Background

Falls are the most common cause of incident reports in long-term care facilities and hospitals [1-3]. Falls can result in physical and psychological trauma; even mortality and increased costs due to prolonged hospital stays [1,4,5].

Many risk factors are identified as contributing inpatient falls. Impaired mental function, impaired mobility, and old age are common denominators associated with the risk of falling. Other fall risk factors are a history of falls, special toileting needs, and medication that targets the central nervous system [6].

The goal of rehabilitation is often to improve locomotion and increase independence. As locomotion improves and a patient becomes more autonomous, the risk for falling also increases. After a fall, it is common to develop a fear of falling [7]. Patients who develop a fear of falling are more likely to have poor outcomes in rehabilitation and at follow-up [7,8]. It is therefore of the utmost importance to acknowledge changes in fall risk status so that the fall prevention is simultaneous with promotion of increased locomotion and independence.

Few studies have shown that falls can be prevented among frail older people in hospitals [9]. Intervention programs to prevent falls can be categorized into single, multiple, and multifactorial. Single and multiple interventions are one or several interventions delivered to all participants in the study. Multifactorial interventions are combinations of interventions tailored after a fall risk assessment that result in a different intervention for each participant [9]. A randomized controlled study among hip fracture patients showed that a multifactorial intervention aiming to prevent postoperative complications such as delirium and falls significantly reduced falls in a treatment versus a control group. The intervention was based on employing systematic routines and staff observations, leading to individualized treatment and compensation for the increased fall risk. The control group was provided with usual care routines [10].

There are numerous scales for evaluating fall risk among older persons that can help staff prevent falls [6]. These scales often consist of a list of fall risk factors that are tallied. The summed result places the patient in one of two or three risk categories, depending on a pre-set threshold value. Many of the scales have been criticized for low sensitivity, reliability, and validity [6,11,12]. Some of them have been compared to a subjective global rating by staff [2,13-15]. These studies showed that staff are able to predict whether or not a person is going to fall at the same level or better than a scale. Therefore, staff judgment can be seen as a “fine-tuned” instrument.

However, there is a lack of knowledge about how staff who are the main caretakers, together with other personnel, make a subjective assessment of fall risk and how they manage fall prevention. The aim of this study was to describe licensed practical nurse experiences of predicting and preventing further falls when working with patients who had experienced a fall-related fracture.

## Methods

### Study design

We conducted a qualitative study based on focus groups and field observations with licensed practical nurses (LPNs) from a university hospital in northern Sweden. The focus group methodology was chosen because of the dynamic way thoughts and experiences are acquired by a group conversation [16]. The interviews were complemented with field observations to facilitate interpretation of the interview data and to increase trustworthiness of the results.

The study was approved by The Regional Ethical Review Board in Umeå (Dnr 2010/322-31M) and by the heads of the clinical departments.

### Setting and participants

Two wards were strategically selected because of previous participation in a fall prevention intervention study [10]. Both wards treated patients directly after the surgery for fall-related fractures. One was a geriatric rehabilitation ward and the other an acute orthopedic ward (Table 1). The staff in both wards treated patients with a history of falls and had some education in fall prevention. However, they worked in different contexts and patient safety efforts were more prominent at the rehabilitation ward.

**Table 1:** Characteristics of the two wards from which the licensed practical nurses were selected

	**Geriatric rehabilitation ward**	**Orthopedic ward**
**Ward layout**	- Single and double rooms.	- Single, double and triple rooms.
	- 24-bed ward, extra beds when needed.	- 24-bed ward, extra beds when needed.
**Patients**	- 65 years or older, many with multiple diseases, the majority with osteoporotic fractures such as hip fractures. Admitted acutely or referred from another clinic.	- Mixed ages, patients with different diagnoses. Any type of fractures, including osteoporotic and high energy fractures, patients with tumors and arthritis. Admitted acutely or planned admissions.
**Mean inpatient stay**	- 24 days	- 6 days
**Teamwork and individual care planning**	- Reports at the start and end of shifts with nurses and licensed practical nurses.	- Reports at the start and end of shifts with nurses and licensed practical nurses.
	- Systematic assessment of the patient by all team members (registered nurses, licensed practical nurses, physical therapists, occupational therapists, dietician and geriatricians) as soon as possible after admittance.	- No team conferences or individual care planning on a routine basis.
	- Team conferences twice a week to monitor patient rehabilitation process and goals.	- Geriatric consults.
	- Orthopedic consults	
**Prevention and treatment of complications**	- Actions to prevent falls and fractures implemented including global ratings and screening tools.	- No systematic routines to prevent falls.
	- Systematic prevention, detection and treatment of postoperative complications.	- No systematic check-ups for postoperative complications.

The work assignments of LPNs include helping the patients with a variety of tasks they cannot manage themselves in daily care. This can for example include serving food, making the bed, cleaning and serving medicine as well as a qualitative dimension. To be a licensed practical nurse does not require graduating studies in Sweden. To be a registered nurse graduating studies is necessary.

The LPNs were strategically selected because they had experience in fall prevention and were believed to be key informants and conversational. In each focus group we aimed for representation of both women and men with different durations of work experience. The selection was made by one of the authors after conferring with the head of each ward. All participants received oral and written information and gave informed consent prior to participation.

The focus groups were stratified by ward. Three interviews were conducted at the geriatric rehabilitation ward (one with night staff), and two at the orthopedic ward. Three licensed practical nurses were in each group. Twelve women and three men completed the focus groups. No one declined participation. Participant age ranged from 30 to 65 years (mean 44.5). Their occupational experience as LPN ranged from 2 to 37 years (mean 23.7).

There were four physical therapists and one psychologist in the research group. Some had extensive experience within the research area of fall prediction and prevention while others had no experience. Thus, the interview information could be looked at with experienced and fresh eyes. Some members of the group had expertise in learning organizations and qualitative research methodology.

### Data collection

One-hour interviews took place in a conference room at the informants’ work unit. All but one informant underwent the interviews during or in connection with their work hours. Compensation was given when interviews were held outside work hours.

**Figure 1:**
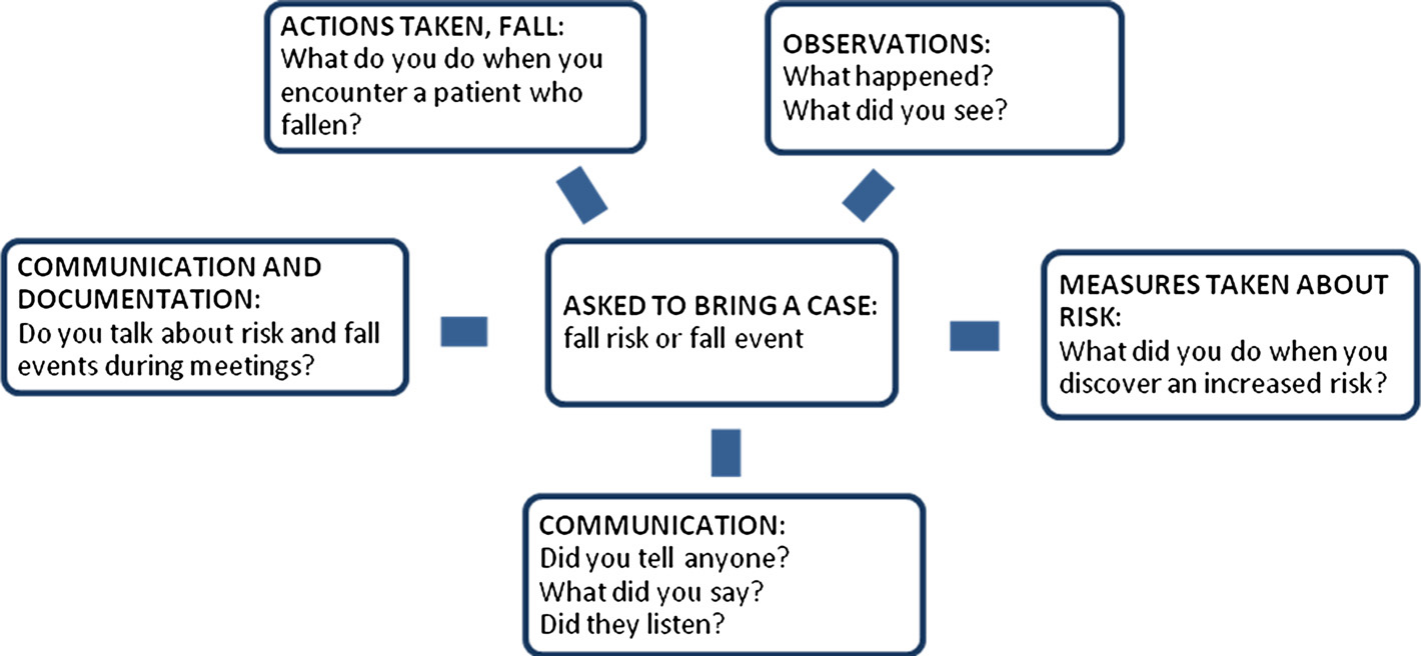
Focus group guide.

The interviews were conducted by a moderator and based on a focus group guide with open questions about observations, preventative measures and communication regarding risk of falling and fall prevention (Figure 1). The focus group guide was discussed and revised by the research group at the beginning of the study and between each interview.

Each focus group discussion started with informant stories about cases they had experience with and had been asked to bring with them. During the interview, the moderator’s role was to distribute the opportunity to speak and maintain a sociable climate since focus group interviews rely on communication and interaction between the participants [16]. An observer was also present during the interviews and was given the opportunity to complement the moderator with follow-up questions. The interviews were audio-recorded and transcribed verbatim.

Field observations were performed in both of the wards. During one day on each ward, the first author observed a LPN performing dayshift activities from early morning to late afternoon. The observer withdrew and took field notes that were based on the LPN actions taken regarding falls and fall prevention. The field notes were included in the data collection.

### Data analyses

The analysis was carried out by qualitative content analysis that expresses the material through comparisons for similarities and differences described in categories and themes [17]. Four members of the research group started with independent, naïve reading of the interviews to get a broad picture and understanding of the context. Each interview was selected as a unit of analysis. Then, the material was read on a more profound level and meaning units were selected from the unit of analysis and codes were created. Meaning units are sentences with rich meaning that speaks of the purpose of the study. Codes are labels for the selected meaning units. The result of the coding was negotiated within the research group to assert the credibility of the material.

**Figure 2:**
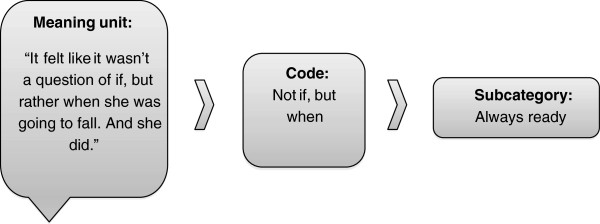
The process of transforming a meaning unit in to a subcategory by qualitative content analysis.

The manifest content of the text, ie, the spoken content in the codes was first sorted into nine subcategories and then formed into three categories (Figure 2). The sorting process of codes was performed by the first author and repeatedly reflected upon and discussed by all the members of the research group. A theme was then identified from the underlying content of the material. Coding and analyses were facilitated by the qualitative data software Open Code (http://www.phmed.umu.se/enheter/epidemiologi/forskning/open-code/).

## Results

**Figure 3:**
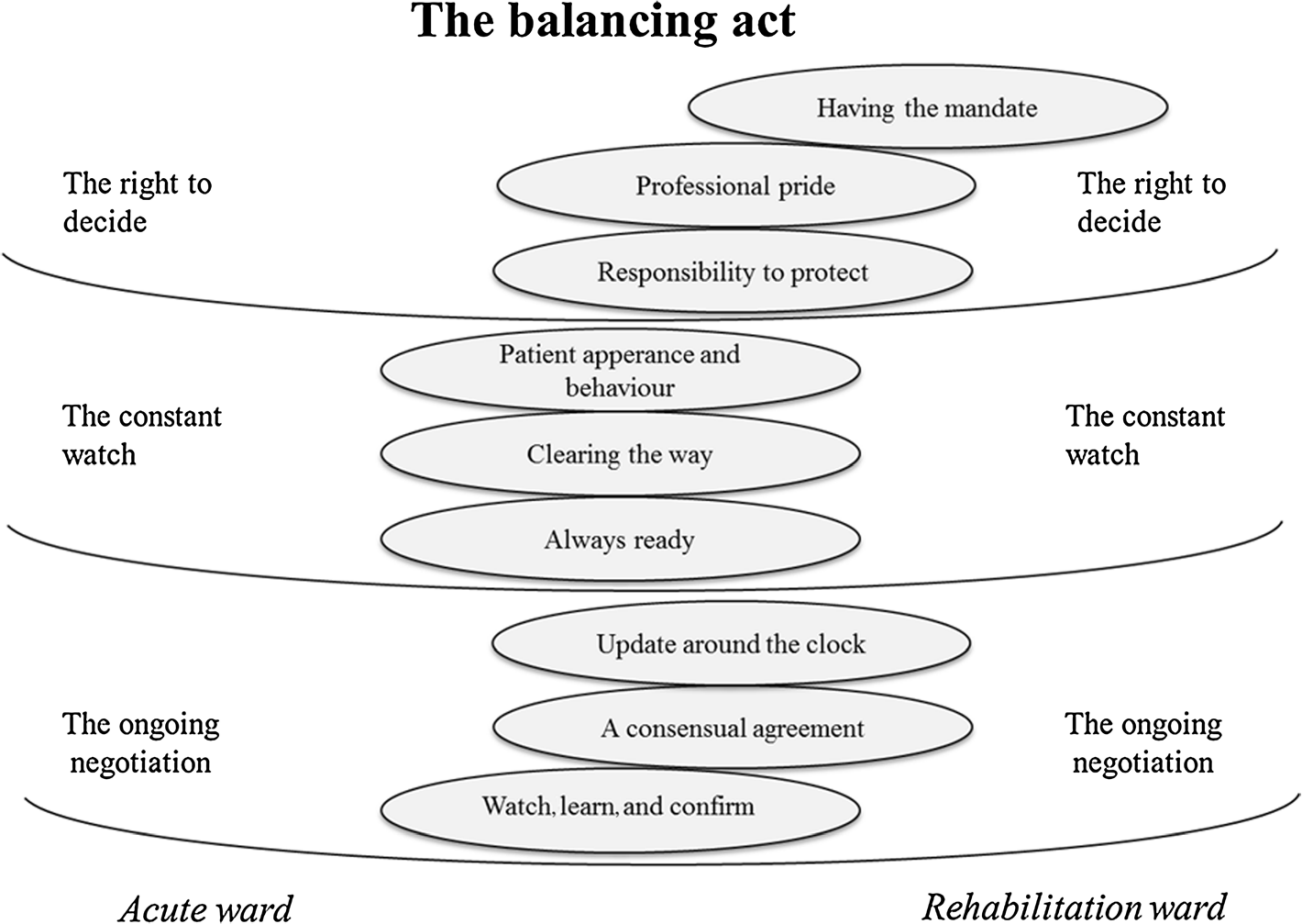
**A model illustrating licensed practical nurse experiences of falls and fall prevention.** Each subcategory, illustrated by an ellipse, is shifted towards the ward that most strongly represented the subcategory.

The licensed practical nurse experiences about fall risk and fall prevention work are presented in one theme: “the balancing act”. The theme includes three categories, “the right to decide”, “the constant watch”, and “the ongoing negotiation”, plus nine subcategories (Figure 3). The analysis showed similarities and differences between acute and rehabilitation wards. The result is illustrated through descriptive quotations in italics. The subcategories are interwoven in the descriptions.

### The right to decide

#### Having the mandate

In the rehabilitation ward, LPNs felt that their leadership had certain expectations of their work regarding fall risk and fall prevention measures. The task of systematically assessing and discussing these issues was considered a work assignment. Every team member was supposed to judge a patient’s risk of falling, and the LPNs were expected to assess each patient’s risk of falling within 48 hours after admission. They were encouraged to voice their opinions and suggest prevention measures.

In the acute ward, the LPNs said the focus was to medically stabilize, mobilize and transfer the patients to another facility or home as soon as possible. They did not experience clear expectations that they were to work systematically with fall prevention, and they did not use the words “risk of falling”.

"“It (fall risk and prevention assessment) is supposed to be done within a certain number of hours. It’s on the work list and looks like a red traffic light. But it increases awareness enormously.” (Interview 1, Rehabilitation ward)"

#### Professional pride

LPNs in both wards took pride in their professional knowledge and were confident in their opinions and assessments. They could speak their minds without the back up of another profession. They considered it important to be able to take a stance and have one’s own initiative.

The LPNs at the rehabilitation ward had learned to see and think of fall risk prevention. Therefore, observations were superior for oral reports and scoring of fall-risk prevention tools. They felt that the knowledge was a part of themselves, almost like *“software in our heads”*.

"“..Can I just step forward and have an opinion? And now we can see that I’m actually right. We know these things! I think that was the biggest hurdle to pass… to dare to write it!” (Interview 1, Rehabilitation ward)"

#### Responsibility to protect

To feel engaged in their patients’ mental and physical health was described as essential by the licensed practical nurses at both wards. Even though the LPNs understood the physical distress that the patient was under, they knew they had to push the patient and sometimes nag. They considered it important to help the patient proceed in the rehabilitation process. Sometimes they worried how a patient at high risk for falls would manage at home.

Patient safety was described as the number one priority, but some elements of that responsibility made them uneasy. At both wards, use of physical restraints caused an emotional conflict. They described the restraints as a double-edge sword because while the restraints took away patient freedom of movement, they temporarily protected the patient from falling, and thereby gave staff a momentary feeling of security. There were also constant reflections upon patient need for support and walking aids. The LPNs made watchful, small successive changes in the support while the patient was transferring or walking. This was a careful process due fear of making a misjudgment. They described it as being overprotective because they did not want to make an error. They were glad when someone else could take on the responsibility for changes regarding the patient. Even though they knew they had done their very best, the LPNs in the rehabilitation ward described emergence of questions such as *“Why wasn’t I there?”* and *“Why didn’t I get there in time?”* when a patient fell.

"“I have to say that I experience it as a failure, when the patient falls.”(Interview 5, Rehabilitation ward)"

### The constant watch

#### Patient appearance and behavior

When LPNs at either ward met patients, they noticed how they walked and acted, and whether they looked ill. Anxious and delirious patients received close attention because the own feelings of uneasiness were considered a warning sign.

The LPNs expressed that they were often observant of how the patient used a walking aid. Patients with poor management of a walker were described as those who forgot the aid, did not properly understand the use of it, or sought other support such as furniture or passersby. One type of walking aid might be sufficient during the day, but not at night. Patient status could also fluctuate throughout the day. Therefore, the risk of falling was seen as something valid here and now, but that could easily change.

High risk of falling was sometimes associated with patient personality. Patients included those who did not know their limitations, did not have the patience to be careful, or took risks by leaving the bed without support and against recommendations. Patients with dementia were described as more vulnerable and in need of extra attention. However, they were not automatically classified as a high fall risk because of this diagnosis.

The trauma of the accident that caused the fracture, and the unfamiliarity to the hospital environment were described as factors leading to disorientation and could lead to an increased risk of falling. This was not seen as a permanent condition. The LPNs explained that it was as if the brain needed to attune, and patients had to get used to the injury itself. Patients could quickly get better after the surgery and be ‘*a whole new person’*.

"“It feels like the risk of falling is precarious. Right after the operation there’s a greater risk, and as days go by it can improve or, just the opposite, take a turn to the worse.” (Interview 2, Rehabilitation ward)"

#### Clearing the way

Licensed practical nurses at both wards had the same opinion about the lack of space for patients. The rehabilitation that was conducted meant a lot of walking aides and wheelchairs and they felt this drastically decreased space in room of the ward. The environment at the ward was perceived as a risk factor for falling even for patients who walked independently.

This problem was counterbalanced by making the bed so that the cover would not constitute a fall risk factor, removing unnecessary walking aids, and clearing the path to the bathroom. Clothes on the walking-aid or on the floor were cleared up as an undeclared routine during the day and at the start of each shift. At the rehabilitation ward, the LPNs described the fall prevention work as a mental checklist that was done routinely and without any thought.

"“You turn around one last time before heading out of the room and sort of check-up.…(think)‘good’, and then you leave.” (Interview 2, Rehabilitation ward)"

#### Always ready

To be always ready was described as a core strategy in licensed practical nurse work. They had to be quick with assessments and make swift decisions. Immediately after surgery the patient had a risk of fainting, could be confused, and in a lot of pain. The LPN also had to pay extra attention to patients who had changes in medication. The LPNs were never completely relaxed and wanted to be near the patients in order to watch them closely.

The LPNs could sometimes, but not always, anticipate the patient’s risk of falling. Both movement pattern and behavior alerted them, and in some cases, they thought that it was not a question of *if* the patient would fall, but *when*.

"“Wow, I would never have anticipated that she would leave her bed, because she looked absolutely relaxed and without pain. And then it was one of those surprises to find her there, fallen down and all. And even getting up with that kind of fracture.…” (Interview 4, Acute ward)"

### The ongoing negotiation

#### Update around the clock

LPNs at both wards reported that oral and written reports were given at the start and end of the work shift, but the content of the reports differed by the ward. At the rehabilitation ward, the routine was to report activities of daily living and issues regarding falls. At the acute ward, they systematically reported diagnoses and blood status, etc. Information was passed on when personnel met each other in the ward rooms or corridors. By these two methods and written reports, there was a constant update of the patient’s status and the information spread quickly.

The LPNs in the rehabilitation ward took for granted that each one knew what the words “risk of falling” meant and used this specific wording. In the acute ward, however, the LPNs felt that “risk of falling” was hard to describe, and they did not use the specific wording. Instead, documentation noted other items like unsteady gait or “dizzy and pale”. When a fall occurred, the immediate measures at both wards were to calm the patient and examine the body. In the rehabilitation ward, they discussed the issue that occurred with each other and began a process of visualizing the patient’s movement pattern and whole situation to tailor a solution that would keep the patient from falling again, and documented the results. In the acute ward, a fall was reported but no discussion followed of how to prevent another. Perhaps they raised a red flag within themselves to be observant, but they “*would not put up a warning sign”*.

"“.. but what I especially remember was that we really put our brains into it…we studiously visualized the movement pattern, how she lies in her bed and how she moves when she sneaks out without notifying.” (Interview 1, Rehabilitation ward)"

#### A consensual agreement

The licensed practical nurses felt it was important that the team had the same patient goals, in order to give uniform care and rehabilitation. Although this was described as important, some LPNs did not follow the consensus decisions and sometimes there were communication difficulties with the physicians. Some perceived the inexperienced staff members to be a burden. On the other hand, some LPNs thought that the inexperienced staff could more easily verbalize the measures taken to prevent falls because it had not yet become routine work to them. Sometimes the many different opinions and experiences among the staff, as well as the views of relatives, made it challenging to reach a consensus. The rehabilitation ward facilitated this through a team forum for talking about risk of falling. The LPNs felt that the team conferences made it possible to exchange ideas concerning patient observations and conclusions. With the team conferences, they could more easily think and discuss each individual patient; they identified this as a chance to make their competence visible.

"“….I think most of it emerges at the team conferences. A lot appears when you’re discussing the patient….Then it all wraps up.” (Interview 2, Rehabilitation ward)"

#### Watch, learn, and confirm

The LPNs compared their own work strategies with their peers and took inspiration to develop from each other. They also copied other LPN roles with patients when these proved to be more efficient than their own. This was not something that was spoken out aloud during daily work, but a quiet learning process.

The LPNs communicated regularly with each other and other members of the team, and felt heard by the others. They anchored their thoughts and assessments regarding patients with nurses and physical therapists, and felt a moral endorsement when they were confirmed.

Some expressed receiving satisfactory confirmation from the manager, but others felt that they did not receive this. They felt that they pressed for certain ideas for years, but were not heard when they wanted a change. This led to irritation and a feeling of a lack of influence.

"“.…He does things in another order. I can see that it flows, it looks safe, and it looks good. When we approach the patient the next time, I can recall how he did things and kind of assume his role.” (Interview 1, Rehabilitation ward)"

### The theme - the balance act

The underlying meaning in the categories was the licensed practical nurse work expressed as a constant balancing act; they not only had to meet administration expectations, but also those of their peers, the patients, and themselves. The LPNs felt they had the responsibility to protect patients from falling and that they did the best they could with the means provided. They emphasized that they had a lot to learn and advance regarding their knowledge about risk of falling.

## Discussion

A culture of patient safety is believed to exist when a common understanding regarding patient safety emerges among staff [18]. The culture is constructed by a dynamic interaction within the organizational structure and formed through two intertwined procedures: 1) reinforcement of reciprocal, multi-professional interactions that include patients and leadership, and 2) a goal-directed facilitation to achieve an understanding of what should be changed and how to do it [19]. The wards included in our study were in different phases of fall prevention implementation. The rehabilitation ward had successfully implemented a multi-factorial and multi-professional intervention program. The fall risk assessments were a task that engaged the whole team, communication was efficient, and leadership and ward infrastructure facilitated the work of preventing falls. Each of these are important parts of a patient safety culture [19]. The acute ward used only a part of the reciprocal phase and had not managed to proceed to the next phase.

The LPN’s right to decide meant that many choices had to be made, often within moments. Previous studies have shown that licensed practical nurses feel a lack of professional status and that their competence is not utilized or adequately acknowledged, nor is the opportunity to express opinions facilitated [20]. This contrasts with our study, where the LPNs expressed a strong professional pride and made it clear that speaking one’s mind was important. The LPNs in the rehabilitation ward were also confident in their ability to judge who was at risk of sustaining a fall. When someone fell, they made tailored changes for that patient. At the acute ward, they had more of a medical focus. If a fall occurred, no patient specific changes were made nor were others alerted of the patient’s fall risk. Outcomes such as fall reporting and less restraints use by LPNs in nursing homes are linked to well-functioning patient safety cultures [21].

We found that mandates for LPNs differed depending on the type of ward where they worked. At the acute ward there was no forum for discussing the issues of fall prediction and prevention among professions and the LPNs felt they lacked words to communicating risk of falling. However, when asked, they could identify fall risk factors that are associated with risk of sustaining a fall. The LPNs in the rehabilitation ward, as a part of their work assignments, were required to complete fall risk assessments to determine who was at risk of falling. They often helped each other describe how the patient moved and behaved. In this way, they created a dialogue and mutual language. Fall risk assessments were routinely discussed at multi-professional meetings that occurred twice a week. The meetings were reported as a significant forum for exchanging information about patient risk of falling. Differences between wards in structure and cultural conditioning may contribute to different ways of working with falls and fall prevention. Many authors emphasize group communication, dialogue, planning, and reflecting as basic ingredients in workplace learning. The team is suggested to have a central role in hospital and long-term care settings [22,23] as well as in other organizations [24]. This is in line with the statements from the LPNs at the rehabilitation ward who learned and transferred knowledge when attending the multidisciplinary meetings. In comparison, in the acute ward, the team had no central role and therefore it might be more complicated to facilitate learning regarding falls. The LPN knowledge about fall risk and fall prevention actions was never articulated and transferred to others.

Quick and constant changes in patient status make it necessary for continuous communication between team members and this often means that there needed to be mediation. To be able to participate in the mediation, LPNs felt it was important to make one’s own assessment. This was also important in order to provide information to one’s peers. There are tools that can be utilized to help LPNs perform fall risk assessments. The acute ward had not implemented a risk assessment tool and did not routinely discuss risk of falling during their meetings. In a study that compared communication during multidisciplinary meetings in two wards, different communication patterns were seen. One ward used a fall risk assessment tool and the other one who did not. This resulted in different patterns in decision-making. Partly this outcome was due to the use of the fall risk assessment tool that helped to create a common language among the professionals [25].

In order for learning to be effective, there must be different opportunities for learning within the healthcare organization. Examples include good communication, opportunities for the staff to participate in decision making, and clear organizational goals [26]. To create a safety culture in a nursing home setting, factors as stress level, work climate, efficient work process, and clear organizational goals have been identified [27]. The wards in our study differed in structure. The rehabilitation ward had a clear goal concerning falls and fall prevention whereas the acute ward had many other goals. Studies in primary care settings have shown that learning structures must be designed and implemented at the workplace, otherwise daily clinical routines becomes the priority [28]. The patient safety culture is significantly associated with management commitment to the issue [29] and steps toward implementation have to come from the organizational level [27].

### Methodological discussion

The research group consisted of researchers with experience in the area of fall prevention, learning organizations, and qualitative research methodology. This strengthened the study by allowing the information given by the LPNs to be understood and interpreted from different angles.

We acknowledge that the low number of participants in the focus groups can be considered a limitation. The recommended lower limit is four participants, and in our study, the number was three [16]. However, prior to this study, three participants had so much they wanted to tell us that we were concerned a larger group would result in a limited time for participants to speak.

A risk when using focus group methodology is that the participants will be influenced by more dominant participants or will only say what is considered socially acceptable [16]. The methodology can also open up discussions and reflections among the participants, and that is what we experienced. The presence of a moderator and observer ensured that a positive climate was maintained and that everyone was allowed to voice their opinions. Achieving both homogeneity and heterogeneity is preferable for focus group composition [16]. In our groups, homogeneity was ensured by a shared work place. We endeavored to achieve heterogeneity by including both men and women, as well as varying years of experience. The goal of heterogeneity was not achieved in three of the focus groups. Those groups lacked men and one group did not have differing years of experience. However, all participants were carefully chosen and considered key stakeholders.

We did not define a fall when conducting the focus groups. Also, during the focus groups, we did not ask how they defined a fall. This is a limitation of our study that could have an impact on our results as a fall can be defined in a number of ways [30]. However, the participants had previously been educated on fall prevention and worked on wards where falls are common. Hence, they should have had some understanding of the definition of a fall.

## Conclusion

Licensed practical nurses work most closely with the patient. When it is expected, they can judge patient risk of falling, share their observations with others, and take actions to prevent falls. The climate and the structure of the ward are essential if licensed practical nurses are to be encouraged to routinely consider risk of falling implement risk reduction strategies. The organization has a valid and resourceful tool in licensed practical nurses for fall prevention, but this is not always fully utilized. Licensed practical nurses are important team members within an organization that aims to develop a patient safety culture.

## Competing interests

The authors declare that we do not have any financial or non-financial competing interests.

## Authors' contributions

BH collected data through the interviews and field studies, analyzed data, and drafted the manuscript. MS participated in the design of the study and in the development of the interview guide, collected data through the interviews, analyzed data, and commented on drafts of the manuscript. AFW participated in the design of the study and in the development of the interview guide, analyzed data, and commented on drafts of the manuscript. KW interpreted and commented on drafts of the manuscript. LLO had the initial idea, participated in the design of the study and in the development of the interview guide, analyzed data, and commented on drafts of the manuscript. All authors have read and approved the final manuscript.

## Pre-publication history

The pre-publication history for this paper can be accessed here:

http://www.biomedcentral.com/1471-2318/12/62/prepub
